# History of foster care among homeless adults with mental illness in Vancouver, British Columbia: a precursor to trajectories of risk

**DOI:** 10.1186/s12888-015-0411-3

**Published:** 2015-02-26

**Authors:** Michelle L Patterson, Akm Moniruzzaman, Julian M Somers

**Affiliations:** Faculty of Health Sciences, Simon Fraser University, 8888 University Drive, Burnaby, British, Columbia Canada

## Abstract

**Background:**

It is well documented that a disproportionate number of homeless adults have childhood histories of foster care placement(s). This study examines the relationship between foster care placement as a predictor of adult substance use disorders (including frequency, severity and type), mental illness, vocational functioning, service use and duration of homelessness among a sample of homeless adults with mental illness. We hypothesize that a history of foster care predicts earlier, more severe and more frequent substance use, multiple mental disorder diagnoses, discontinuous work history, and longer durations of homelessness.

**Methods:**

This study was conducted using baseline data from two randomized controlled trials in Vancouver, British Columbia for participants who responded to a series of questions pertaining to out-of-home care at 12 months follow-up (n = 442). Primary outcomes included current mental disorders; substance use including type, frequency and severity; physical health; duration of homelessness; vocational functioning; and service use.

**Results:**

In multivariable regression models, a history of foster care placement independently predicted incomplete high school, duration of homelessness, discontinuous work history, less severe types of mental illness, multiple mental disorders, early initiation of drug and/or alcohol use, and daily drug use.

**Conclusions:**

This is the first Canadian study to investigate the relationship between a history of foster care and current substance use among homeless adults with mental illness, controlling for several other potential confounding factors. It is important to screen homeless youth who exit foster care for substance use, and to provide integrated treatment for concurrent disorders to homeless youth and adults who have both psychiatric and substance use problems.

**Trials registration numbers:**

Both trials are registered with the International Standard Randomized Control Trial Number Register and were assigned ISRCTN57595077 (Vancouver At Home Study: Housing First plus assertive community treatment versus congregate housing plus supports versus treatment as usual) and ISRCTN66721740 (Vancouver At Home Study: Housing First plus intensive case management versus treatment as usual) on September 9, 2012.

## Background

The childhoods of homeless adults are disproportionately characterized by persistent poverty, residential mobility, school problems, and other stressful and/or traumatic events [1,2], particularly among homeless individuals with mental illness [3]. One of the earliest identifiable precursors to homelessness may be placement in out-of-home or foster care. Studies of youth “aging out” of the foster care system indicate that between 11% and 36% [4,5] experience street homeless and approximately one-third live with family, friends or acquaintances because they cannot afford permanent housing [6]. In fact, many youth are discharged from foster care without a place to live [6].

In addition to challenges around finding stable housing, many youth exiting foster care do not complete high school and have difficulty finding employment [7,8]. Underlying difficulties related to the transition to adulthood among youth leaving foster care is a cumulative history of trauma, abuse and neglect that results in a number of neurobiological and behavioural deficits including poor social support and coping skills [9,10] as well as risk for further abuse and victimization [8]. Once homeless or “drifting”, street culture promotes antisocial behavior, including association with deviant peers and means of subsistence (e.g., sex trade, theft, drug dealing) [11]. The cumulative impact of these factors places youth at high risk for a wide range of health and psychosocial problems including substance misuse and chronic homelessness [12].

A large body of evidence suggests that adverse childhood experiences, which typically include physical, sexual, and emotional abuse, neglect, dysfunctional family environments, and unstable family structure, are linked to later biological, psychological and social functioning and may affect multiple domains of health and well-being [12-14]. Moreover, it appears that adverse childhood experiences tend to cluster together [15,16] and the *number* of adverse experiences may be more predictive of negative adult outcomes than particular categories of events. Tsai, Edens and Rosenheck [17] examined the childhood profiles of 738 homeless adults and found three clusters: numerous childhood problems (21%); disrupted family structure (44%); and few childhood problems (35%). Participants with histories of foster care were significantly more likely to fall into the ‘numerous childhood problems’ category, which in turn predicted younger age when first homeless and more severe drug use than other participants. Tam, Zlotnick and Robertson [18] examined the long-term effects of adverse childhood experiences on adult substance use, social service use, and employment among homeless adults in Oakland. Adverse childhood experiences were positively correlated with consistent substance use over 15 months of follow-up as well as social service use. Further, consistent substance use was negatively associated with employment and social service use. However, this latter study did not separate foster care from other adverse childhood events, therefore, the contribution of foster care to various outcomes is not clear.

More specifically, research has documented the association between childhood foster care placement and subsequent homelessness [7,11]. Further, research with homeless populations has found that childhood histories of foster care are significantly correlated with psychiatric disorders in adulthood [3,19]. Roos et al. [19] examined the association between foster care history and a range of health and social outcomes among homeless adults with mental disorders, 75% of whom were Aboriginal, in Winnipeg, Canada. Compared to participants without histories of foster care, those placed in care were significantly more likely to be young, female, less educated, married/cohabitating, Aboriginal, and reported a longer lifetime duration of homelessness. A history of foster care independently predicted a range of adult outcomes including depression, psychosis, substance dependence and interpersonal violence. While various forms of interpersonal violence were examined, different types and frequencies of substance use were not included. A more in depth examination of the impact of foster care on various aspects of substance use is required.

In this study, we focus on the relationship between foster care placement as a predictor of adult homelessness and associated health and service outcomes among a sample of homeless adults with mental disorders. Specifically, we examine the relationship between foster care history and adult substance use (including age of initiation, frequency, severity and type of substance), mental illness, vocational functioning, service use and duration of homelessness. We hypothesize that a history of foster care predicts earlier, more severe and more frequent substance use, multiple mental disorder diagnoses, discontinuous work history and longer durations of homelessness.

## Methods

The Vancouver At Home Study includes two randomized controlled trials involving homeless adults with mental illness in Vancouver, British Columbia. Vancouver At Home is one of five experimental projects investigating Housing First in Canada, funded by the Federal Government [20]. Details related to the trials including eligibility criteria, recruitment, randomization, retention, measures, interventions, and hypotheses have been reported elsewhere [21]. The current study focuses on baseline data from both trials in Vancouver and does not incorporate any longitudinal findings. The two trials are distinguished based on participants’ level of need. Based on an algorithm that included information about diagnosis, community functioning, and service use, participants were assigned to a “high” or “moderate ” need group [20,21].

Eligibility criteria included legal adult status (19 years and older), current mental disorder on the MINI International Neuropsychiatric Interview (MINI) [22], and being absolutely homeless or precariously housed. The MINI is a structured diagnostic interview that can be administered by non-clinicians and inquires about symptoms related to the following categories: Psychotic Disorder, Mood Disorder with Psychotic Features, Hypo(manic) Episode, Major Depressive Episode, Suicide Risk, Panic Disorder, Post-traumatic Stress Disorder, Alcohol Dependence, and Substance Dependence. Absolute homelessness was defined as living on the streets or in an emergency shelter for at least the past seven nights with little likelihood of obtaining secure accommodation in the upcoming month. Precariously housed was defined as living in a rooming house, hotel or other transitional housing; in addition, individuals must have experienced at least two episodes of absolute homelessness in the past year, or one episode lasting for at least four weeks in the past year.

Participants were recruited through referral from over 40 agencies available to homeless adults in Vancouver; the majority was recruited from homeless shelters, drop-in centers, homeless outreach teams, hospitals, community mental health teams, and criminal justice programs. Referral was typically initiated by service providers and a preliminary screening for eligibility (e.g., duration of homelessness, mental health and substance use problems), was conducted via telephone with the referral agent. All participants met face-to-face with a trained research interviewer who explained procedures, obtained informed consent, and confirmed study eligibility. A cash honorarium of $5 was provided to the participant for the screening process. Institutional ethics board approval was obtained through Simon Fraser University and the University of British Columbia. Data derived through the shared cross-site protocol are owned by the study sponsor (Mental Health Commission of Canada). Approval for use of baseline data from both trials used in this study was granted by the study sponsor. Data that are specific to the Vancouver site (site-specific instruments, administrative data) are retained at the host institution (Simon Fraser University).

If the individual met all study criteria, they were enrolled as a participant and the baseline interview commenced, consisting of a series of interviewer-administered questionnaires including socio-demographic characteristics, psychiatric symptoms, substance use, physical health, service use, and quality of life [21]. Participants received a further cash honorarium of $30 upon completion of the baseline interview, which typically took 90 minutes to complete. The following analyses are based upon data from the baseline questionnaires of 497 participants recruited from October 2009 to June 2011 and data from a series of questions related to out-of-home care, which were administered 12 months after baseline.

### Variables of interest

Details associated with out-of-home care were elicited by a series of questions related to personal experiences of out-of-home care and having one’s biological children placed in out-of-home care. First, participants were asked if they ever lived away from their parents prior to age 18 and, if so, the reason for living away from home. Second, participants were asked if they were ever placed in foster care, the reason for the placement, and their age and the duration of the most recent placement. Finally, participants were asked if they had biological children currently or previously placed in foster care or other forms of out-of-home care.

Mental disorders were assessed using the MINI and were grouped, based on convention [23], into a *severe* cluster, comprised of (at least one) current Psychosis, Mood Disorder with Psychotic Features, and Hypo(manic) Episode, and a *less severe* cluster comprised of Major Depressive Episode, Panic Disorder, and Post-traumatic Stress Disorder (PTSD). Frequency and type of substance use over the past month were recorded using the Maudsley Addiction Profile (MAP) [24], a structured interview which inquires about a wide range of substances and the number of days used in the past month, the amount used on a typical day, and the route of administration. Physical illness was assessed by self-report using a checklist of 30 chronic health conditions (lasting longer than six months). Blood-borne infectious disease consisted of positive self-report diagnosis of HIV, Hepatitis B or Hepatitis C. Vocational functioning was assessed via two questions: (1) have you ever had a job that lasted for at least one year? (yes/no) and (2) are you currently employed in paid work? (yes/no) Psychometric properties for all measures are provided in previous manuscripts [20,21].

### Statistical analyses

Descriptive statistics (median and inter-quartile range for continuous variables; frequency and percentage for categorical variables) were used to characterize the sample. All continuous variables included in statistical analyses (i.e., age at randomization, duration of homelessness, age first homeless, age of first drug/alcohol use) were transformed into categorical variables. For dichotomous variables, median values were used as the cut-off point. Comparisons of categorical data between participants who completed or did not complete the foster care items, and those who did or did not report a history of foster care, were conducted using Pearson’s chi-square test.

Univariate and multivariable logistic regression analyses were used to model the independent associations between placement in foster care and a series of outcome variables that were established a priori based on previous literature [6,8,25,26]. Each outcome variable was modeled in both univariate and multivariate settings using placement in foster care as an independent risk factor. Outcome variables that were significant at the p ≤ 0.05 level were considered for the multivariable logistic regression analyses using the same set of controlling variables (i.e., age at randomization, gender, ethnicity, marital status, and level of need), chosen based on previous literature [11]. Both unadjusted and adjusted odds ratios and 95% confidence intervals (CI) are reported as effect sizes and all p-values are two-sided. IBM SPSS Statistics (version 22) was used to conduct the analyses. Missing values that ranged from zero to 1% for all outcome and controlling variables in the regression analysis were excluded, with the exception of age of first alcohol and drug use. A small minority of participants who reported never using alcohol and/or drugs did not have valid information for these variables (5% and 9% respectively).

## Results

In total, 497 participants completed the baseline questionnaires. Of the total sample, 449 participants (90%) were located for the 12 month interview and 442 of those participants provided a valid response when asked if they had ever been placed in foster care. Among 442 participants, only 5 (1%) responded “Don’t know” which was categorized as “No”. An additional 1% of participants declined to respond to the foster care questions and was excluded from the analysis. Table 1 presents the baseline characteristics for the full baseline sample (n = 497) and for participants who completed all of the foster care items (n = 442). At baseline, the majority of participants who responded to the foster care items were male (72%) and White (57%); the mean age at randomization was 41.0 (SD = 10.9) years; and the mean age when first homeless was 30.1 (SD = 13.4) years. The median duration of lifetime homelessness was 36 months (IQR: 12–84 months). Compared to participants who did not complete the foster care items (n = 55), those who did were more likely to engage in early alcohol use (before age 14) and less likely to report weekly alcohol use in the month prior to enrollment (p ≤ 0.05). Otherwise, there were no significant differences at baseline between participants who completed vs. did not complete the foster care items.

**Table 1:** **Socio**-**demographic**, **mental disorder**, **and drug**-**use related characteristics for Vancouver At Home participants** (**n** = **497**)

Variable	All participants (n = 497) N (%)	Participants with valid response ( n = 442) N (%)	Participants with missing response (n = 55 ^ 1 ^ ) N (%)	P value
Need level (high)	297 (60)	267 (60)	30 (55)	0.403
Housing first interventions	297 (60)	274 (62)	23 (42)	0.004**
Male gender	359 (73)	317 (72)	42 (76)	0.531
Age at randomization				
19-24 years	36 (7)	29 (7)	7 (13)	0.198
25-44 years	281 (57)	254 (57)	27 (49)	
>44 years	180 (36)	159 (36)	21 (38)	
Ethnicity				
Aboriginal	77 (15)	68 (15)	9 (16)	0.951
White	280 (56)	250 (57)	30 (55)	
Other	140 (28)	124 (28)	16 (29)	
Incomplete high school	280 (57)	249 (57)	31 (56)	0.960
Single marital status	343 (70)	305 (69)	38 (73)	0.562
Have children (under age 18)	122 (25)	109 (25)	13 (24)	0.919
Hospitalized for mental illness (>6 months) in past 5 years	57 (12)	51 (12)	6 (11)	0.855
Hospitalized for mental illness (>2 times) in past 5 years	253 (53)	222 (52)	31 (60)	0.291
Worked continuously at least one year in the past	323 (65)	285 (65)	38 (69)	0.540
Unemployed	457 (92)	406 (92)	51 (93)	0.905
Jail in last 6 months	68 (14)	57 (13)	11 (20)	0.148
Precariously housed	109 (22)	96 (22)	13 (24)	0.822
Duration of homelessness - lifetime (>36 months)^2^	234 (48)	211 (48)	61 (42)	0.357
Duration of homelessness - longest single period (>12 months)	245 (50)	222 (51)	23 (42)	0.203
Age of first homelessness (<25 years)	214 (44)	192 (44)	22 (40)	0.569
Overall health (poor)	67 (13)	60 (14)	7 (13)	0.857
Less severe mental illness	264 (53)	235 (53)	29 (53)	0.951
Severe mental illness	363 (73)	323 (73)	40 (73)	0.956
Multiple (≥2) mental disorders	240 (48)	213 (48)	27 (49)	0.900
Alcohol dependence	121 (24)	104 (23)	17 (31)	0.229
Substance dependence	288 (58)	258 (58)	30 (55)	0.588
High suicidality	87 (17)	79 (18)	8 (14)	0.540
Blood-borne infectious diseases	157 (32)	142 (32)	15 (28)	0.490
Multiple (≥3) physical illness	344 (69)	309 (70)	35 (64)	0.342
Early initiation of alcohol (≤13 years)	215 (46)	198 (47)	17 (32)	0.037*
Early initiation of drugs (≤13 years)	183 (40)	167 (41)	16 (30)	0.096
Daily substance use	143 (29)	127 (29)	16 (29)	0.956
Daily drug use	126 (25)	113 (26)	13 (24)	0.756
Daily illicit drug use^3^	74 (15)	69 (16)	5 (9)	0.200
Injection drug use	88 (18)	83 (19)	5 (9)	0.076
Use of multiple (≥2) substances	257 (52)	231 (53)	26 (47)	0.454
Use of multiple (≥2) drugs	188 (38)	173 (39)	15 (27)	0.081
Daily use of alcohol	26 (5)	23 (5)	3 (5)	0.949
Daily use of cannabis	70 (14)	61 (14)	9 (16)	0.606

*p ≤ 0.05.

**p ≤ 0.01.

^1^Among 55 participants with missing values, 7 participants declined to respond and 48 participants could not be located for the 12 months interview.

^2^Dichotomized based on median value (3 years).

^3^Does not include alcohol and marijuana.

As shown in Table 2, 59% of participants reported that they lived away from their parents prior to age 18. The primary reasons for living away from home were placement in foster care (37%) and running away from home (25%). Thirty percent of participants reported being placed in foster care as a child, primarily due to parental abuse and/or neglect (38%). The median number of foster care placements was 1.0, the median age of the most recent placement was 10 years, and the median duration of the most recent placement was 18 months. Further, 9% of participants reported having children currently in foster care and 23% reported having children in foster care in the past (see Table 2).

**Table 2:** **Foster care experiences among Vancouver At Home participants**

	N (%)
Lived away from parents before age 18 (n = 445)	264 (59)
Reasons for living away (n = 264)^4^	
Lived with relatives or friends	36 (14)
Foster home	98 (37)
Group home	23 (9)
Jail or detention	18 (7)
Ran away or kicked out	75 (28)
Moved out	28 (11)
Other	36 (14)
Placed in foster care (n = 442)	135 (30)
Reasons (n = 135)^5^	
Parental illness or accident	19 (14)
Parental incarceration	5 (4)
Parental abuse or neglect	54 (40)
Behavioural problems	34 (25)
Don’t know	19 (14)
Other	14 (10)
Number of foster care placements (n = 125)	
Median (IQR)	1 (1–4)
Age at time of most recent placement (n = 131)	
Median (IQR)	10 (3–13)
Duration (months) of most recent placement (n = 122)	
Median (IQR)	18 (8–48)
Biological children (currently or in the past) in foster care (n = 447)	41 (9)
Biological children (currently or in the past) adopted or living with someone else (n = 446)	105 (23)

^4^Proportions were derived using 264 as the denominator. Participants could endorse more than one reason.

^5^Proportions were derived using 135 as the denominator. Participants could endorse more than one reason.

Bivariate comparisons by foster care history (yes/no) are summarized in Table 3. Participants with histories of foster care were significantly more likely to share a particular socio-demographic profile (i.e., female, Aboriginal ethnicity, incomplete high school, younger age at enrollment, have children under age 18, no continuous work history); they also first experienced homelessness at a younger age (before 25 years) and, due to their younger age, reported shorter durations of homelessness (both lifetime and longest single episode). Participants with histories of foster care were significantly more likely to meet criteria for: the less severe cluster of mental disorders (i.e., major depressive episode, panic disorder, PTSD); two or more mental disorders; substance dependence; high suicide risk; early initiation of alcohol and/or drug use (before age 14); current daily drug use; injection drug use; and poly-drug use.

**Table 3:** **Comparisons of socio**-**demographic**, **mental disorders and drug**-**use related characteristics by history of foster care among Vancouver At Home participants** (**n** = **442**)

Variable	Foster care - no	Foster care - yes	P value
Need level			
High	186 (70)	81 (30)	0.908
Moderate	121 (69)	54 (31)	
Study arm			
Housing first (HF)	190 (69)	84 (31)	0.947
Treatment as usual (TAU)	117 (70)	51 (30)	
Gender			
Male	231 (73)	86 (27)	0.011*
Female	73 (60)	48 (40)	
Age at randomization			
19-24 years	18 (62)	11 (38)	0.007**
25-44 years	164 (65)	90 (35)	
>44 years	125 (79)	34 (21)	
Ethnicity			<0.001***
Aboriginal	26 (38)	42 (62)	
White	188 (75)	62 (25)	
Other	93 (75)	31 (25)	
Educational attainment			<0.001***
Completed high school	152 (80)	38 (20)	
Incomplete high school	153 (61)	96 (39)	
Marital status			0.880
Single (never married)	213 (70)	92 (30)	
Other	94 (69)	42 (31)	
Have children (under age 18)			0.001***
No	237 (73)	87 (27)	
Yes	62 (57)	47 (43)	
Worked continuously at least one year in the past			<0.001***
No	89 (58)	65 (42)	
Yes	215 (75)	70 (25)	
Unemployed			0.346
No	26 (77)	8 (23)	
Yes	279 (69)	127 (31)	
Jail (past 6 months)			0.085^+^
No	273 (71)	112 (29)	
Yes	34 (60)	23 (40)	
Housing status			0.357
Precariously housed	63 (66)	33 (34)	
Absolutely homeless	244 (71)	102 (29)	
Duration of homelessness - lifetime			0.012*
>36 months^6^	168 (75)	57 (25)	
≤36 months	134 (64)	77 (36)	
Duration homeless - longest single episode			0.001***
>12 months^7^	164 (77)	50 (23)	
≤12 months	138 (62)	84 (38)	
Age of first homelessness			<0.001***
≥25 years	192 (79)	52 (21)	
<25 years	111 (58)	81 (42)	
Overall health			0.944
Fair/good/excellent	265 (70)	116 (30)	
Poor	42 (70)	18 (30)	
Less severe mental illness			0.001***
No	160 (77)	47 (23)	
Yes	147 (63)	88 (37)	
Severe mental illness			0.879
No	82 (69)	37 (31)	
Yes	225 (70)	98 (30)	
Multiple (≥2) mental disorders			<0.001***
No	176 (77)	53 (23)	
Yes	131 (62)	82 (38)	
Alcohol dependence			0.954
No	235 (70)	103 (30)	
Yes	72 (69)	32 (31)	
Substance dependence			0.011*
No	140 (76)	44 (24)	
Yes	167 (65)	91 (35)	
Suicide risk			0.034*
High	260 (72)	103 (28)	
No/low/moderate	47 (60)	32 (40)	
Blood-borne infectious diseases			0.730
No	207 (70)	89 (30)	
Yes	97 (68)	45 (32)	
Multiple (≥3) physical illness			0.415
No	96 (72)	37 (28)	
Yes	211 (68)	98 (32)	
Initiation of alcohol			<0.001***
Before 14 years	173 (78)	48 (22)	
14 years or after	115 (58)	83 (42)	
Initiation of drugs			<0.001***
Before 14 years	179 (76)	57 (24)	
14 years or after	93 (56)	74 (44)	
Frequency of substance use			0.005**
Less than daily/none	231 (73)	84 (27)	
Daily	76 (60)	51 (40)	
Frequency of drug use			0.003**
Less than daily/none	241 (73)	88 (27)	
Daily	66 (58)	47 (42)	
Injection drug use			0.003**
No	257 (73)	95 (27)	
Yes	47 (57)	36 (43)	
Poly-substance use			0.012**
Two or less	157 (76)	51 (24)	
Three or more	149 (65)	82 (35)	
Poly-drug use			0.007**
Two or less	198 (74)	68 (26)	
Three or more	108 (62)	65 (38)	
Frequency of alcohol use			0.348
Less than daily/none	291 (70)	124 (30)	
Daily	14 (61)	9 (39)	
Frequency of cannabis use			0.057^+^
Less than daily/none	271 (71)	110 (29)	
Daily	36 (59)	25 (41)	

*p ≤ 0.05.

**p ≤ 0.01.

***p ≤ 0.001.

^+^p > 0.05 & p ≤ 0.10.

^6^Dichotomized based on median value (3 years).

^7^Dichotomized based on median value (1 year).

Unadjusted (UOR) and adjusted odds ratios (AOR) and 95% CI for variables included in the univariate and multivariable analyses are presented in Table 4. Results from the multivariable logistic regression analyses indicate that foster care history independently predicted incomplete high school (AOR: 2.08), duration of homelessness – lifetime (AOR: 1.60) and longest single episode (AOR: 1.95), age of first homelessness (AOR: 2.36), continuous work history (AOR: 0.62); meeting criteria for the less severe cluster of mental disorders (AOR: 1.62) and two or more mental disorders (AOR: 1.69); early initiation of alcohol (AOR: 2.54) and/or drugs (AOR: 2.49) and current daily drug use (AOR:1.64). A significant positive trend (p > 0.05 and p ≤ 0.10) was observed between a history of foster care and meeting criteria for panic disorder (AOR: 1.63), PTSD (AOR: 1.60), high risk of suicide (AOR: 1.59), daily substance use (AOR: 1.53), daily cannabis use (AOR: 1.74), as well as incarceration (AOR: 1.70) and court appearance (AOR: 1.48) in the past six months.

**Table 4:** **Logistic regression analyses for socio**-**demographic**, **mental disorder and substance use related outcomes of foster care experiences** (**n** = **442**)

Outcome Variable ^ 8 ^	Unadjusted OR ( 95 % CI )	P value	Adjusted ^ 9 ^ OR ( 95 % CI )	P value
Incomplete high school	2.51 (1.62, 3.89)	<0.001***	2.08 (1.29, 3.35)	0.003**
**Homelessness**				
Lifetime duration (>3 years)^10^	1.69 (1.12, 2.55)	0.012*	1.60 (1.02, 2.50)	0.041*
Longest single episode (>1 year)^11^	2.00 (1.32, 3.03)	0.001***	1.95 (1.25, 3.05)	0.003**
Age first homeless (<25 years)	2.69 (1.77, 4.10)	<0.001***	2.36 (1.42, 3.91)	0.001***
**Vocational functioning**				
Worked continuously (≥1 year) in the past	0.45 (0.29, 0.68)	<0.001***	0.62 (0.39, 0.98)	0.041*
Unemployed	1.50 (0.65, 3.36)	0.349	-	
**Mental disorders**				
Major depressive episode	1.44 (0.96, 2.17)	0.078+		
Panic disorder	1.56 (0.97, 2.51)	0.067+		
Post-Traumatic Stress Disorder	2.22 (1.43, 3.47)	<0.001***	1.60 (0.98, 2.62)	0.061^+^
Less severe cluster	2.04 (1.34, 3.10)	0.001***	1.62 (1.03, 2.57)	0.039*
(Hypo) manic episode	1.25 (0.76, 2.06)	0.374		
Mood disorder with psychotic features	1.21 (0.72, 2.04)	0.478		
Psychotic disorder	0.69 (0.46, 1.04)	0.078+		
Severe cluster	0.97 (0.61, 1.52)	0.879	-	
Alcohol dependence	1.01 (0.63, 1.63)	0.954	-	
Substance dependence	1.73 (1.13, 2.65)	0.011*	1.18 (0.74, 1.88)	0.501
High suicide risk	1.72 (1.04, 2.84)	0.035*	1.59 (0.92, 2.72)	0.095^+^
Multiple mental disorders (≥2)	2.08 (1.38, 3.14)	0.001***	1.69 (1.08, 2.66)	0.023*
**Physical health**				
Blood-borne infectious diseases	1.08 (0.70, 1.66)	0.730		
Multiple physical illness (≥3)	1.21 (0.77, 1.89)	0.415		
Overall health (poor)	0.98 (0.54, 1.77)	0.944		
**Substance use**				
Early initiation of alcohol (<14 years)^12^	2.60 (1.70, 3.99)	<0.001***	2.54 (1.60, 4.05)	<0.001***
Early initiation of drugs (<14 years)^13^	2.50 (1.63, 3.83)	<0.001***	2.49 (1.56, 3.96)	<0.001***
IV drug use	2.07 (1.27, 3.40)	0.004**	1.50 (0.87, 2.59)	0.141
Daily substance use	1.85 (1.20, 2.85)	0.006**	1.53 (0.96, 2.44)	0.077^+^
Daily drug use (no alcohol)	1.95 (1.25, 3.05)	0.003**	1.64 (1.01, 2.65)	0.045*
Daily illicit drug use^14^	1.70 (1.00, 2.89)	0.050*	1.23 (0.69, 2.19)	0.490
Daily alcohol use	1.51 (0.64, 3.58)	0.351		
Daily marijuana use	1.71 (0.98, 2.98)	0.058+		
Poly-substance (≥2) use	1.69 (1.12, 2.57)	0.013*	1.22 (0.77, 1.94)	0.393
Poly-drug (≥2) use (no alcohol)	1.75 (1.16, 2.65)	0.008**	1.32 (0.84, 2.09)	0.232
**Justice System Involvement**				
Incarceration (past 6 months)	1.65 (0.93, 2.93)	0.087+		
Been arrested (past 6 months)	1.41 (0.92, 2.15)	0.114		
Court appearance (past 6 months)	1.50 (0.98, 2.28)	0.061+		

*p ≤ 0.05.

**p ≤ 0.01.

***p ≤ 0.001.

^+^p > 0.05 & p ≤ 0.10.

^8^Separate binary logistic regression analyses (univariate and multivariable) were conducted for each outcome using Foster Care (No vs. Yes) as an independent variable.

^9^Controlled for age (continuous), gender (Male, Female), ethnicity (Aboriginal, White, Other), need level (High, Moderate), and marital status (Single, Other).

^10^Dichotomized based on median value (3 years).

^11^Dichotomized based on median value (1 year).

^12^Dichotomized based on median value (14 years).

^13^Dichotomized based on median value (14 years).

^14^Does not include alcohol and marijuana.

## Discussion

Among our sample of homeless adults with mental illness, we found a significant relationship between a history of childhood foster care placement and a number of indicators of socio-demographic risk, poor mental and physical health, and problematic substance use in adulthood. Foster care history independently predicted incomplete high school, duration of homelessness, discontinuous work history, meeting criteria for a current mental disorder in the less severe cluster, multiple mental disorders, early initiation of alcohol and/or drug use, and daily drug use. Our study is the first to examine a range of different adult substance use outcomes (i.e., type, frequency, poly-substance use, age of initiation) among homeless adults with mental illness and histories of foster care placement.

Foster care placement independently predicted substance use problems in our adult sample, specifically early initiation (before age 14) of drug and/or alcohol use and daily drug use. Abuse of alcohol and other drugs places adolescents at greater risk of homelessness, although it may not be a direct causal factor [27]. Compared to their non-homeless counterparts, homeless youth use substances earlier and with greater frequency [28,29]. Further, Thompson and Hasin [30] reported that homeless youth with histories of foster care were almost nine times more likely to have been in drug treatment than homeless youth without such histories, even after adjusting for demographics, other adverse childhood events, prior arrest, unemployment and educational attainment.

Our findings suggest that daily drug use may be a common mediator for a range of early risk factors [31]. Previous research using our sample of homeless adults with mental disorders found that daily drug use was independently predicted by self-report of childhood learning disabilities [32] and adverse childhood experiences [33]. In the latter study, we found a strong relationship between the breadth of exposure to abuse or household dysfunction during childhood (i.e., the *number* of adverse childhood events) and a number of indicators of poor mental and physical health as well as problematic substance use in adulthood (e.g., daily drug use). In turn, previous work with the same sample has shown that daily drug use significantly predicts the duration of adult homelessness [34] and the severity of mental health symptoms [35].

The current study did not examine other potential mediators such as self-esteem or shame; however, one could assume that strong manifestations of these psychological states might manifest in the mental disorders examined in our study. Daily drug use and other potential mediators (e.g., emotion regulation skills, access to social support and early intervention, antisocial behaviors) need to be examined in future studies using path-type analysis. Cross-sectional, retrospective data cannot disentangle the unique predictors of homelessness and mental illness, but it is likely that adverse childhood experiences, particularly foster care placement, have both direct and indirect (perhaps mediated by substance use) effects on a range of adult health and social outcomes. Thus, among homeless adults with mental disorders, a history of early and persistent substance abuse may be a key indicator of risk. Our findings support the need for more accessible and integrated care for homeless adults with concurrent disorders.

In addition to substance use problems, a history of foster care independently predicted meeting criteria for a less severe type of mental disorder (i.e., major depressive episode, panic disorder, PTSD) and more than one mental disorder. It makes sense that foster care placement would contribute to mood and anxiety disorders more so than psychosis. However, Roos et al. [19] found that a history of foster care predicted adult psychotic disorders as well as depression and multiple mental disorders. While the disorders comprising our less severe cluster may appear less serious than those in the severe cluster, the disorders in the former cluster can be very debilitating. The fact that foster care predicts meeting criteria for multiple mental disorders suggests that poor psychiatric outcomes in adulthood are related to foster care, and further strengthens the case for integrated treatment for concurrent disorders for this population.

Given that over 1,000 youth exit foster care without a family each year in Canada, services and supports need to be in place to help youth address concurrent psychiatric and substance use problems prior to and after discharge. Organizations that provide services to homeless youth should target those with histories of adverse childhood events, particularly foster care, and tailor services to their collective and individual needs. Approaches such as brief motivational interviewing have been shown to reduce substance use among homeless youth in a variety of settings [36,37] and should be made more widely available as part of integrated treatment for concurrent disorders.

The significant association between foster care history and the duration of homelessness in adulthood is also of concern. Both researchers and advocates have called for more evidence-based interventions to reduce homelessness among youth exiting foster care [8,11]. Preliminary research on transitional living programs for youth exiting foster care is associated with reduced rates of street homelessness and increased employment, particularly when paired with vocational skills training [38].

In previous work, we have emphasized the importance of viewing homelessness in the context of cumulative adversity [39] and Problem Behavior Theory [40,41]. These models conceptualize homelessness as the result of successive environmental disruptions, each of which places individuals at greater risk for homelessness and associated risk factors. In addition to the effects of progressive risk, risk may be multiplied at each stage of development because the environmental risks associated with later phases present increasingly greater challenges to development and adaptation. Problem Behavior Theory hypothesizes that various early risk factors may comprise a cluster of risky behaviors that mediate the link from childhood adversity to a variety of adverse adult outcomes, rather than distinct independent pathways. Our findings, alongside others’, suggest that the relationship between foster care placement and adult homelessness (and the associated health and social problems we identified) may be mediated by problem behaviours such as heavy substance use. Further, it is likely that the number of adverse events (e.g., adverse childhood events in addition to foster care, incomplete high school, early problems with substance use and/or the law, lack of institutional or family support) may be more important in predicting negative adult health and social outcomes than any one event. Daily or heavy substance use must be tested as a mediating factor alongside other potential mediators.

Placement in out-of-home care is a significant and salient event that should indicate appropriate interventions for both children and parents. Early interventions in childhood might change or moderate the cycle of homelessness across generations because early risk factors are often longstanding and drive a trajectory of cumulative risk, potentially leading to severe psychopathology and social exclusion. Among our sample of homeless adults, 31% had children currently or previously in foster care, suggesting that foster care placement is a form of trauma that has inter-generational effects.

### Limitations

Despite the strengths of our study design (i.e., large sample size, structured diagnostic interviews), several limitations must be considered. First, all variables were based on participant self-report. Given that participants were selected based on presence of a current mental disorder, accuracy of recall may have been compromised. Further, the baseline interview occurred before randomization to a housing intervention and therefore may have led some participants to modify their responses. It is unlikely that participants purposefully modified their responses to the foster care items given that they were administered after the baseline interview. Furthermore, foster care is a highly salient event that is likely to be remembered; however, recall error could have occurred in questions pertaining to the reason for being placed in care.

Additionally, data were not available to examine whether participants’ biological mothers had abused substances during pregnancy, the age participants first entered foster care, the total length of time they spent in foster care, and number and types of residential and educational placements. As each of these factors has been shown to increase the risk for subsequent psychiatric disorders, the effect of these factors on the risk for psychiatric and substance use disorders should be examined in future research. In addition to the above noted variables, we did not have access to several other important variables necessary for conducting a robust path analysis in order to more fully investigate the links between foster care placement and adverse outcomes among homeless adults (e.g., early problem behaviors, social support, affect regulation skills, self-esteem/self-concept, and the availability of treatment/institutional supports). Future longitudinal studies should examine foster care in the context of other adverse childhood events, early problem behaviors that have been identified in the literature (e.g., adolescent substance use, involvement with the law, incomplete high school), as well as key adult outcomes, in order to investigate the key pathways that link foster care to adverse outcomes among homeless adults with mental illness.

## Conclusion

This is the first Canadian study to investigate the relationship of a history of foster care with current substance use among homeless adults with mental illness, controlling for several potential confounding factors. The high rates of foster care history observed among our sample of homeless adults with mental illness highlight the dire consequences that can result from this trajectory of risk. Given the salience of removing children from the family home and placing them in foster care, this should be a sentinel event that triggers a range of interventions aimed at preventing negative adult psychiatric, health and social outcomes.
